# Stress-induced pacemaker desynchronization in the sinoatrial node

**DOI:** 10.3389/fcvm.2026.1852965

**Published:** 2026-06-23

**Authors:** Dong-Gyun Han

**Affiliations:** Han's Neurology Clinic, Daejeon, Republic of Korea

**Keywords:** autonomic perturbation, defervescence, orthostatic autonomic loading, pacemaker synchronization, sinoatrial node, sleep-stage transition, sympathovagal coactivation, synchronization reserve

## Abstract

The sinoatrial node (SAN) functions as a heterogeneous population of electrically coupled pacemaker cells rather than as a single dominant oscillator. We propose a SAN-centered hypothesis in which stress-related arrhythmogenic vulnerability may arise from transient loss of synchronization within this pacemaker population. Sustained autonomic stress, recurrent orthostatic autonomic loading associated with human upright posture, inflammatory remodeling, fibrosis, or structural SAN remodeling may amplify intrinsic-frequency dispersion and reduce effective coupling, thereby lowering synchronization reserve. Abrupt autonomic, respiratory, or thermal transitions, including stress-recovery sympathovagal transitions, sleep-related respiratory events, fever-associated thermal acceleration, and rapid defervescence, may then expose this vulnerable substrate. In this framework, arrhythmogenesis reflects not only abnormal impulse generation, conduction disturbance, reentry, or afterdepolarization-mediated triggered activity, but also perturbation-induced loss of pacemaker-network coherence and transient competition among pacemaker domains. The model remains a reduced phenomenological framework rather than a calibrated anatomical simulation, but it yields testable predictions, including transition-linked sinus cycle-length instability, supraventricular ectopy, *P*-wave morphology variability, and reduced coherence in heterogeneous SAN models. This hypothesis suggests that attention should also be directed to dynamic synchronization reserve during physiological challenge, rather than resting rate alone.

## 1 Introduction

The sinoatrial node (SAN) serves as the primary pacemaker of the heart, but its rhythmic output does not arise from a single immutable oscillator. The SAN is a structurally and functionally heterogeneous tissue composed of pacemaker cells and pacemaker regions that differ in intrinsic automaticity, ion-channel expression, calcium-handling dynamics, gap-junctional coupling, and electrotonic interaction with the surrounding atrium (1–5). Classical electrophysiology explains SAN function through membrane currents, intracellular calcium cycling, conduction, and intercellular coupling (1–3). These mechanisms remain essential for normal pacemaking and rhythm disturbance, yet they do not fully describe episodic instability that appears during abrupt transitions in physiological state.

Clinical and experimental observations indicate that arrhythmias often emerge during changes in autonomic, respiratory, circadian, or thermal state rather than during stable baseline conditions alone. Psychological stress increases arrhythmic vulnerability through heart-brain and autonomic pathways, and stress-related electrophysiological instability has been documented in susceptible individuals (6–8). An additional human-relevant substrate-forming context is habitual upright posture. The human-relevant vulnerability proposed here does not require the human SAN to be fundamentally different from that of other mammals. Rather, habitual upright posture may have placed a conserved heterogeneous mammalian SAN substrate under recurrent orthostatic autonomic loading (1–5, 9). Over evolutionary and lifetime timescales, this loading may increase the propensity for sympathovagal overlap or autonomic overshoot-recovery dynamics, thereby lowering synchronization reserve and increasing pacemaker-dispersion vulnerability in susceptible individuals (10–13). This upright-posture component is therefore treated as chronic or recurrent autonomic loading that may condition the substrate, not as an abrupt perturbation window. Sleep introduces repeated autonomic and respiratory transitions, while circadian variation in sudden cardiac death underscores the contribution of time-dependent physiological state to arrhythmic risk (14, 15). Temperature also modulates pacemaking biology, because body temperature contributes to circadian regulation of sinoatrial nodal pacemaking and HCN4 channels participate directly in heart-rate responses to heat (16, 17). These observations support a complementary mechanism in which rhythm instability reflects disturbed coordination within the pacemaker system, not only altered excitability of individual cells.

This perspective motivates a systems-level reformulation of SAN function. The SAN can be modeled as a heterogeneous population of coupled pacemaker cells whose collective behavior depends on synchronization (1–5). Under stable conditions, coupling allows this population to generate coherent sinus rhythm despite substantial cellular heterogeneity (1–5). Sustained autonomic stress, chronic inflammatory remodeling, structural SAN remodeling, or altered thermal responsiveness may enlarge internal dispersion in pacemaker behavior before overt arrhythmia becomes apparent (3, 5–8, 14–17). The relevant pathological variable is therefore not only mean firing rate, but erosion of synchronization reserve within the pacemaker population.

Stress-related arrhythmogenesis can be framed as an interaction between substrate and perturbation. The substrate consists of amplified heterogeneity across SAN pacemaker cells or domains, which may be shaped by sustained autonomic loading, recurrent orthostatic autonomic loading associated with habitual upright posture, chronic inflammation, fibrosis, or structural SAN remodeling. The perturbation consists of an abrupt autonomic, respiratory, or thermal transition imposed on this vulnerable substrate. Thus, chronic remodeling and recurrent upright-posture-related autonomic loading are treated as substrate-forming processes rather than as acute triggers. When intrinsic-frequency dispersion becomes sufficiently large relative to effective coupling, even a modest perturbation may produce transient loss of coherence, phase competition among pacemaker regions, pacemaker shift, or ectopic escape (4, 5). Stress-recovery transitions, sleep-related transitions, and rapid defervescence are therefore relevant not because they are inherently pathological, but because they may reveal latent instability in a pacemaker population whose internal coordination has already been weakened (14–17).

This article proposes a testable SAN-centered hypothesis: sustained autonomic or stress-related loading, chronic inflammatory remodeling, and structural SAN remodeling may form a vulnerable pacemaker substrate, while abrupt physiological perturbations expose transient synchronization failure. The model complements established ionic, conduction-based, and triggered-activity frameworks by focusing on population-level coherence of the primary pacemaker network. In this view, the SAN operates not merely as a rate-generating structure but as a dynamically coordinated oscillator population whose loss of internal synchrony may create arrhythmogenic vulnerability (1–9, 14–17).

## 2 The sinoatrial node as a heterogeneous oscillator network

The heterogeneity of the SAN has been recognized for decades. Regional differences in morphology, action potential configuration, ion-channel expression, calcium-handling properties, gap-junction distribution, and pacemaker responsiveness have all been documented across the node (1–5). More recent work has further shown that pacemaking is not fixed to a single anatomical point; the leading pacemaker site can shift within the SAN, and distinct superior, central, and inferior pacemaker regions may dominate under different physiological conditions (4, 5). Sympathetic stimulation tends to shift the dominant pacemaker superiorly, whereas parasympathetic stimulation tends to shift it inferiorly (4, 5). These findings support the view that normal sinus rhythm emerges from a dynamically organized pacemaker population rather than from a single immutable oscillator.

At a conceptual level, this system can be represented as a population of coupled oscillators. Let the phase of pacemaker cell *i* be denoted by $\theta_{i}$ and its intrinsic frequency by $\omega_{i}$. A simplified Kuramoto-type representation may be written as

$$\frac{d\theta_{i}}{dt}=\omega_{i}+\frac{K}{N}\sum_{\;j=1}^{N}\sin(\theta_{j}-\theta_{i}),$$ (1)

where *K* denotes an effective coupling strength and *N* is the number of coupled oscillators. Equation 1 uses a mean-field coupling term to summarize synchronization pressure across the pacemaker population. It should not be interpreted as an anatomical claim that all SAN cells are globally and uniformly coupled; native SAN interactions are local, gap-junctional, electrotonic, regionally heterogeneous, and anisotropic. Equation 1 is therefore a reduced phenomenological representation of synchronization reserve, intended to complement rather than replace ionic, tissue-level, or anatomically resolved SAN models (18–20).

Prior work has already applied oscillator and Kuramoto-type approaches to cardiac pacemaker synchronization and heterogeneous pacemaker tissue (18–20). The novelty of the present framework therefore does not lie in introducing oscillator synchronization into cardiac theory, but in linking SAN heterogeneity to stress-conditioned coherence loss and perturbation-triggered arrhythmogenic vulnerability.

The collective degree of synchronization can be expressed by the standard Kuramoto order parameter. To summarize phase alignment across the pacemaker population, each oscillator *j* is represented as a unit phasor $e^{i\theta_{j}}$, and the population-average phasor is computed as

$$W=\frac{1}{N}\sum_{\;j=1}^{N}e^{i\theta_{j}}=Re^{i\psi },R=|W|,0\leq R\leq 1.$$ (2)

Here, *W* denotes the complex population-average phasor, ψ denotes the mean phase, and R=∣W∣ quantifies the degree of phase coherence of the oscillator population. In Equation 2, $i=\sqrt{-1}$ denotes the imaginary unit, whereas subscripts such as *i* and *j* index pacemaker cells or domains. Because *R* is the magnitude of *W*, it is bounded between 0 and 1. Values close to 1 indicate close phase alignment, whereas lower values indicate increasing phase dispersion. Thus, high *R* corresponds to stable population-level synchronization and coherent sinus rhythm, whereas reduced *R* indicates weakening synchronization reserve. In the present framework, desynchronization is represented operationally by a reduction in *R* as intrinsic-frequency dispersion increases relative to effective coupling.

Under stable conditions, the SAN behaves as a synchronized network despite substantial cellular heterogeneity (1–5). The present framework proposes that sustained autonomic stress does not merely change mean pacemaker rate; it enlarges the dispersion of intrinsic pacemaker behavior across SAN cells. As this dispersion increases relative to effective coupling, synchronization reserve declines and the order parameter *R* decreases. Arrhythmogenic vulnerability therefore arises from impaired coherence within a heterogeneous pacemaker population, not only from altered mean rate.

## 3 Autonomic modulation as a source of pacemaker heterogeneity

Autonomic tone modulates SAN activity through established membrane-clock and calcium-clock mechanisms. Sympathetic stimulation accelerates pacemaking by increasing cAMP-dependent inward currents, enhancing calcium cycling, and steepening phase-4 depolarization, whereas vagal stimulation slows pacemaking through acetylcholine-sensitive potassium currents and related intracellular signaling pathways (2, 3). A compact cell-specific abstraction is

$$\omega_{i}=\omega_{0,i}+\alpha_{i}S-\beta_{i}V,$$ (3)

In Equation 3, $\omega_{0,i}$ is the baseline intrinsic frequency of cell *i*, *S* is sympathetic drive, *V* is vagal drive, and $\alpha_{i}$ and $\beta_{i}$ denote cell-specific sensitivities to these inputs. This expression treats autonomic input not only as a mean-rate modulator but also as a source of differential modulation across SAN cells.

Autonomic modulation need not be spatially or functionally uniform across the SAN. If pacemaker cells differ in receptor expression, intracellular signaling gain, calcium-clock dependence, local metabolic state, or microenvironmental conditions, autonomic activation may alter both the mean intrinsic frequency and the variance of the population (3, 5). Autonomic input can therefore function as a heterogeneity amplifier. The relevant pathological change is widening of intrinsic pacemaker dispersion across the network, not simply acceleration or deceleration of pacemaking.

This point is strengthened by the autonomic-space framework, which treats sympathetic and parasympathetic regulation as a two-dimensional response space rather than a single reciprocal continuum. Four canonical response modes can be distinguished: reciprocal sympathetic activation (S↑, V↓), reciprocal parasympathetic activation (S↓, V↑), sympathovagal coactivation (S↑, V↑), and coinhibition (S↓, V↓) (10, 11). Operational assessment of autonomic balance or coactivation can depend on measurement approach, and impedance-derived cardiac autonomic metrics should therefore be interpreted cautiously (12). The coactivation mode is especially relevant to the present model because recovery from acute stress may begin before sympathetic tone has fully returned to baseline; vagal rebound can therefore overlap with residual sympathetic drive (13).

In a homogeneous pacemaker population, this dual autonomic input would mainly shift mean sinus rate. In a heterogeneous SAN, however, pacemaker domains may differ in both adrenergic gain $\alpha_{i}$ and vagal gain $\beta_{i}$. Residual sympathetic drive may continue to accelerate highly adrenergic domains, while recovering vagal input may preferentially suppress other domains. The result may be a transient widening of intrinsic-frequency dispersion rather than a uniform acceleration or deceleration of the whole SAN. This non-uniform response provides the intuition for the variance formulation in Equation 4. Coactivation is therefore not assumed to be arrhythmogenic by itself; it becomes potentially destabilizing when adrenergic and vagal sensitivities are distributed unevenly across SAN domains. Simultaneous autonomic activation has already been implicated in atrial arrhythmogenesis, and the present hypothesis extends this principle to pacemaker-domain coherence within the SAN (21, 22).

$$\sigma_{\omega }^{2}(t)=\mathrm{Va}r_{i}[\omega_{0,i}+\alpha_{i}S(t)-\beta_{i}V(t)].$$ (4)

Equation 5 gives the key coactivation-sensitive cross-term in the variance expansion:

$$C_{\mathrm{coact}}(t)=-2S(t)V(t)\mathrm{Co}v_{i}(\alpha_{i},\beta_{i}).$$ (5)

This cross-term clarifies why coactivation does not have a single obligatory effect. Because S(t)>0 and V(t)>0 during sympathovagal overlap, a negative covariance between adrenergic and vagal gains makes the coactivation cross-term positive; in that case, the cross-term adds to the frequency-dispersion variance and can widen intrinsic-frequency dispersion. This situation would occur when highly adrenergic pacemaker domains have relatively low vagal gain, while other domains show the opposite pattern. Conversely, a positive covariance makes the cross-term negative and may partially offset dispersion. Thus, the proposed arrhythmogenic factor is not sympathovagal coactivation by itself, but coactivation acting on a heterogeneous SAN with limited coupling reserve.

This broader idea is consistent with the arrhythmia literature linking stress to electrophysiological instability. Psychological stress is increasingly recognized as a driver of arrhythmogenesis through heart–brain interactions and autonomic mechanisms (6). In susceptible patients, laboratory stress has been associated with repolarization instability, and ambulatory monitoring studies have linked self-reported stress to greater ectopic burden (7, 8). Although these studies have largely focused on ventricular rather than sinoatrial phenomena, they support the more general principle that stress can translate into electrophysiological instability through autonomic pathways (6–8). The present hypothesis extends that principle to the primary pacemaker network itself.

The SAN should therefore be understood not as a single homogeneous pacemaker, but as a heterogeneous population of coupled pacemaker regions with different intrinsic rates and differential autonomic responsiveness (1–5). Under stable conditions, these regions remain synchronized enough to function as one dominant pacemaker (1, 2, 4, 5). Abrupt sympathoadrenal activation, stress-recovery sympathovagal coactivation, vagal withdrawal, or vagal rebound may modulate pacemaker domains non-uniformly. This differential response may broaden the internal frequency distribution, create functional pacemaker-domain competition, and increase susceptibility to rhythm instability. Direct demonstration of this process in the human SAN remains a specific experimental target (3–5, 10–13, 21, 22).

## 4 From stress exposure to pacemaker-domain susceptibility

The proposed mechanism consists of two linked stages. The first stage is substrate formation. The SAN is a heterogeneous pacemaker network with differing intrinsic frequencies, ion-channel properties, calcium-cycling states, and coupling states. Its stability depends on the mean intrinsic pacemaker frequency, the degree of heterogeneity across the population, and the strength of effective intercellular coupling.

Within this framework, sustained or repeated autonomic stress increases the dispersion of intrinsic pacemaker behavior across the SAN. This heterogeneity can be summarized by the variance of intrinsic pacemaker frequencies across pacemaker cells or pacemaker domains:

$$\sigma_{\omega }^{2}=\mathrm{Va}r_{i}(\omega_{i}).$$ (6)

Here, $\sigma_{\omega }^{2}$ reflects cell-to-cell or domain-to-domain variability in intrinsic pacemaker frequency around the population mean. Stress is hypothesized to increase $\sigma_{\omega }^{2}$, even if the mean intrinsic frequency also rises under sympathetic drive. The arrhythmogenic substrate is therefore not merely sinus tachycardia, but increased heterogeneity within the pacemaker population.

The resulting coherence of the SAN can be expressed schematically by introducing a phenomenological response function *F*, which maps intrinsic-frequency dispersion and effective coupling strength onto the order-parameter magnitude R:

$$R=F(\sigma_{\omega }^{2},K_{\mathrm{eff}}),\;\frac{\partial F}{\partial \sigma_{\omega }^{2}}\;<0,\;\;\frac{\partial F}{\partial K_{\mathrm{eff}}}>0,\;0\leq R\leq 1.$$ (7)

**Figure 1:**
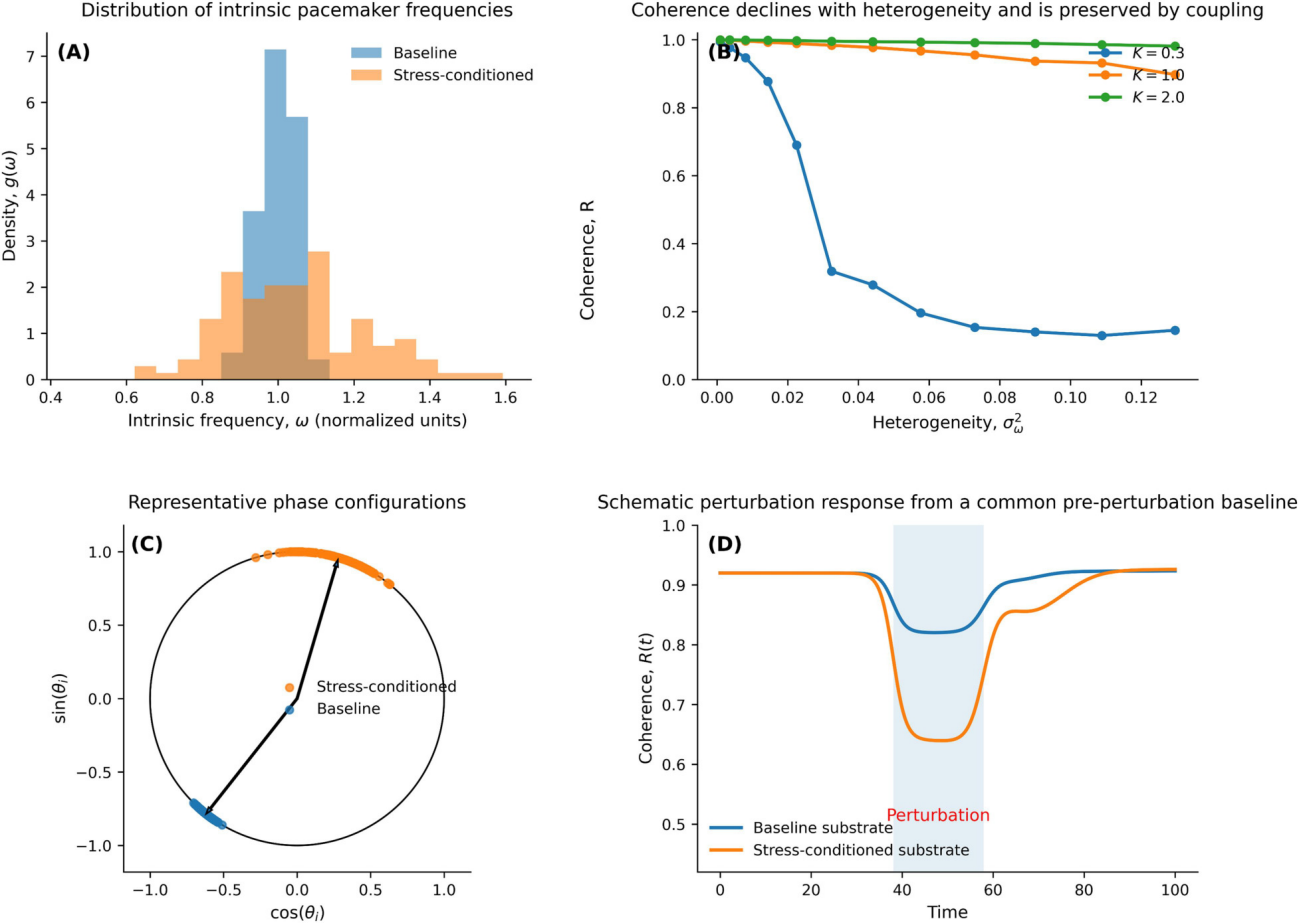
Schematic representation of stress-induced coherence loss in a heterogeneous sinoatrial pacemaker network. **(A)** Baseline and stress-conditioned probability density distributions of intrinsic pacemaker frequencies, f(ω). Stress conditioning broadens the distribution, indicating increased frequency dispersion, with a modest rightward shift representing sympathetic rate acceleration. **(B)** Representative coherence, *R*, as defined in Equation 2, as a function of intrinsic-frequency variance, $\sigma_{\omega }^{2}$, for different effective coupling strengths, $K_{\mathrm{eff}}$. Coherence decreases as dispersion increases and is better preserved when coupling is stronger. **(C)** Unit-circle phase configurations, with each oscillator plotted as the unit phasor $e^{i\theta_{i}}$. Stress conditioning produces greater phase dispersion and weaker collective alignment. **(D)** Schematic perturbation responses from a common pre-perturbation baseline. The stress-conditioned substrate shows a deeper transient fall in coherence, *R*, and slower recovery after perturbation. The panel illustrates the proposed state-dependent response and is not a calibrated biophysical simulation.

Here, *F* is not intended as a fitted biophysical function, but as a phenomenological response function summarizing the expected directional dependence of coherence on dispersion and coupling. *R* is the same order-parameter magnitude defined in Equation 2. The inequalities in Equation 7 indicate that increasing intrinsic-frequency dispersion lowers coherence, whereas stronger effective coupling preserves coherence toward, but not beyond, the upper bound of 1. SAN coherence is therefore determined not primarily by mean pacing rate, but by the balance between heterogeneity, which promotes desynchronization, and coupling, which opposes it. Figure 1 illustrates this balance schematically.

The second stage is perturbation-triggered synchronization failure. Once heterogeneity becomes sufficiently large relative to effective coupling, the SAN may no longer behave as a single coherent oscillator. At this stage, the population distribution of intrinsic frequencies, g(ω), may no longer behave like a single narrow peak. Instead, it may transiently resemble a mixture of relatively slower and faster pacemaker subpopulations:

$$g(\omega )=p\;g_{1}(\omega )+(1-p)\;g_{2}(\omega ).$$ (8)

In Equation 8, $g_{1}(\omega )$ and $g_{2}(\omega )$ denote relatively slower and faster pacemaker subpopulations, respectively, and *p* denotes their relative weighting. The overall pacemaker population is therefore represented conceptually as a weighted mixture of two partially distinct frequency groups rather than as a single narrow peak. This formulation does not claim that stable bimodality has already been demonstrated in living human SAN tissue. It provides a systems-level representation of transient pacemaker-domain competition among regions that differ in intrinsic frequency, autonomic responsiveness, or coupling reserve (4, 5).

In this scenario, the clinically relevant problem is declining internal synchrony rather than elevated sinus rate alone. As coherence weakens, different pacemaker regions may transiently compete for dominance, producing pacemaker shift, unstable cycle length, or ectopic escape from synchronized control (4, 5). The model reframes stress-related rhythm vulnerability as reduced coordination within a heterogeneous oscillator population rather than as a purely rate-based abnormality.

This concept should be distinguished from classical wandering atrial pacemaker. Wandering pacemaker is an ECG-level phenomenon in which the dominant atrial pacemaker shifts sequentially, often producing changing *P*-wave morphologies. The proposed SAN desynchronization model refers to a subclinical or mesoscopic loss of phase coherence within the SAN pacemaker population. Wandering pacemaker may represent one macroscopic manifestation of pacemaker-domain instability, but the model proposed here emphasizes transient fragmentation or simultaneous competition among pacemaker domains before a single dominant exit site becomes apparent on surface ECG.

## 5 Sleep-related autonomic perturbations and SAN synchronization

Sleep provides repeated autonomic and respiratory transitions that may challenge SAN synchronization reserve. Non-REM sleep generally favors vagal predominance, whereas REM sleep is associated with autonomic volatility and ventricular ectopy in susceptible contexts (14). Circadian sudden death literature supports state-dependent cardiovascular vulnerability, but not a specific SAN mechanism (15). Sleep-disordered breathing, intermittent hypoxemia, arousal, and sympathetic surges provide another perturbation route (23). The autonomic-space and sympathovagal-overlap framework described above provides the mechanistic bridge to the present SAN hypothesis (10–13, 21, 22). The present model does not treat REM sleep as the exclusive or primary perturbation; the relevant variable is an abrupt autonomic or respiratory transition acting on a vulnerable pacemaker substrate.

If autonomic inputs vary with time, the intrinsic frequencies of SAN pacemaker cells become time dependent:

$$\omega_{i}(t)=\omega_{0,i}+\alpha_{i}S(t)-\beta_{i}V(t).$$ (9)

In Equation 9, S(t) and V(t) denote time-varying sympathetic and vagal influences. The intrinsic pacemaker tendency of each SAN cell or domain may therefore fluctuate across sleep stages, respiratory events, and stress-recovery intervals. Transient sympathovagal coactivation, vagal withdrawal, arousal-related sympathetic discharge, or hypoxemia-driven autonomic activation may increase cell-specific variability in $\omega_{i}(t)$, thereby increasing the variance term in Equation 6 and reducing coherence as represented schematically in Equation 7. In a robustly synchronized SAN, such forcing may alter sinus rate and shift the leading pacemaker site without overt rhythm instability. In a vulnerable SAN with enlarged heterogeneity, the same perturbation may reduce *R*, permit phase competition among pacemaker regions, and facilitate supraventricular ectopy or escape activity.

Accordingly, the cited sleep and circadian literature should be interpreted cautiously. Some evidence concerns ventricular ectopy or sudden cardiac death rather than direct SAN desynchronization (14, 15). These studies support the broader principle that autonomic state transitions can increase arrhythmogenic vulnerability, but they do not prove a sleep-stage-specific SAN mechanism. The most direct predictions of the present model concern SAN-derived or supraventricular phenomena, including sinus cycle-length instability, pacemaker shift, atrial escape, premature atrial complexes, and beat-to-beat *P*-wave morphology variability. REM-rich periods in the latter part of the night may provide one type of perturbation, whereas non-REM hypoxemia or arousal in sleep-disordered breathing may provide another. The relevant variable is the abruptness and magnitude of autonomic or respiratory forcing imposed on a vulnerable pacemaker network, not REM sleep alone.

## 6 Thermal perturbation, kinetic recovery, and inflammation

Temperature provides another route by which SAN synchronization may be perturbed. Heat accelerates heart rate, and cardiac pacemaker mechanisms are temperature sensitive (16, 17). A standard temperature-dependent relationship can be expressed in $Q_{10}$ form as

$$k(T)=k_{0}\;Q_{10}^{(T-T_{0})/10},$$ (10)

where k(T) denotes a temperature-dependent kinetic process, $k_{0}$ is its value at reference temperature $T_{0}$, and $Q_{10}$ is the temperature coefficient, defined as the fold change associated with a 10 °C increase in temperature. The temperature difference $(T-T_{0})/10$ is expressed in degrees Celsius in the physiological $Q_{10}$ convention.

Applying the $Q_{10}$ -type relationship in Equation 10 to intrinsic pacemaker frequency, a simplified cell-specific expression is

$$\omega_{i}(T)=\omega_{0,i}\;Q_{10,i}^{(T-T_{0})/10},$$ (11)

In Equation 11, $\omega_{i}(T)$ is the intrinsic frequency of pacemaker cell or domain *i* at temperature *T*, $\omega_{0,i}$ is its baseline intrinsic frequency at reference temperature $T_{0}$, and $Q_{10,i}$ is allowed to vary across cells. Cell-specific $Q_{10,i}$ captures the hypothesis that SAN pacemaker domains may differ in thermal responsiveness. In physiological applications, $T_{0}$ can be interpreted as a reference body temperature, such as approximately 37 °C.

Direct evidence that fever increases SAN heterogeneity remains lacking. Nevertheless, because the SAN is intrinsically heterogeneous (1) and pacemaker mechanisms are temperature sensitive (16, 17), fever may differentially affect pre-existing pacemaker domains rather than accelerate the entire SAN uniformly. The dominant clinical effect may still appear as sinus tachycardia, reflecting an upward shift in mean intrinsic frequency. In a susceptible network, however, domain-specific thermal gain could widen internal frequency dispersion and weaken coordinated pacemaker hierarchy (1, 24).

Rapid defervescence is best framed as a kinetic recovery problem rather than as a simple reversal of fever-induced tachycardia. The model does not require different macroscopic cooling rates across superior and inferior SAN regions. Instead, it proposes that functionally specialized SAN domains may differ in their recovery kinetics because of differences in ion-channel composition, calcium-clock dependence, autonomic receptor density, metabolic state, inflammatory exposure, or coupling to atrial load. Thus, even under a common systemic temperature trajectory, different SAN domains may not reset their intrinsic frequencies in parallel.

A schematic representation is given in Equation 12:

$$\frac{d\omega_{i}}{dt}=\lambda_{rec,i}[\omega_{i}^{*}(T(t))-\omega_{i}(t)],\;\;\lambda_{rec,i}>0$$ (12)

In Equation 12, where $\omega_{i}(t)$ denotes the current intrinsic frequency of domain *i*, $\omega_{i}^{*}(T(t))$ denotes the temperature-dependent target or steady-state intrinsic frequency toward which domain *i* tends at the current body temperature T(t), and $\lambda_{\mathrm{rec},i}$ denotes the domain-specific thermal recovery rate. If $\lambda_{\mathrm{rec},i}$ differs across SAN domains, intrinsic frequencies may return toward their temperature-dependent targets at different rates during cooling. As a result, transient frequency dispersion can persist even after systemic temperature has begun to normalize. Desynchronization is therefore not an inevitable consequence of defervescence; it is expected only when the resulting frequency dispersion exceeds the available effective coupling reserve.

Inflammatory signaling and structural remodeling provide a complementary substrate mechanism (25–27). Chronic inflammation is not treated here as the same type of fast perturbation as stress-recovery sympathovagal transition, sleep-related respiratory or autonomic transition, or defervescence. Instead, it may modify the baseline substrate by altering HCN4 expression, calcium handling, metabolic state, fibrosis, gap-junction distribution, and SAN–atrium exit properties. In model terms, inflammation may increase intrinsic-frequency dispersion $\sigma_{\omega }^{2}$ and reduce effective coupling $K_{\mathrm{eff}}$, thereby moving the system closer to a desynchronization threshold before an acute perturbation occurs.

$$K_{\mathrm{eff}}=K_{0}-\gamma_{F}F-\gamma_{I}I,$$ (13)

In Equation 13, *F* represents fibrotic or structural remodeling, *I* represents inflammatory signaling, and $\gamma_{F}$ and $\gamma_{I}$ summarize their effects on coupling reserve. This expression is schematic rather than fitted. It clarifies how slow substrate formation and fast perturbation interact: chronic inflammatory or structural SAN remodeling forms a vulnerable substrate by increasing intrinsic-frequency dispersion and reducing effective coupling, whereas an acute autonomic, respiratory, or thermal transition acts on that substrate and reveals the vulnerability.

In most individuals, thermal and inflammatory perturbations remain compensated. In a susceptible SAN with enlarged intrinsic-frequency dispersion or reduced coupling reserve, fever-associated thermal acceleration followed by non-parallel defervescence-associated resetting could weaken synchronization reserve and increase vulnerability to supraventricular ectopy or rhythm instability. Current evidence supports temperature sensitivity of pacemaker mechanisms and an emerging role of inflammatory remodeling in sinus node dysfunction, but direct evidence that rapid defervescence destabilizes SAN synchronization in humans remains lacking (16, 17, 24–27). The value of the hypothesis is that it identifies a concrete physiological transition and kinetic mechanism that can be examined experimentally.

## 7 Clinical implications and testable predictions

Clinically, the model predicts transition-linked SAN instability rather than resting-rate abnormality alone. Risk assessment should therefore pair substrate markers, such as sustained autonomic stress, recurrent orthostatic autonomic loading associated with habitual upright posture, autonomic lability, sleep-disordered breathing, or inflammatory/structural SAN remodeling, with defined perturbation windows, including stress recovery, sleep-stage transition, sleep-disordered breathing events, apnea termination, arousal, fever onset, antipyretic-associated defervescence, or rapid fever resolution.

Specific predictions are: (1) short-window PP or sinus cycle-length instability will increase around perturbation windows; (2) supraventricular ectopy, atrial escape, pacemaker shift, or *P*-wave morphology variability will cluster around those windows; (3) stressed or remodeled subjects will show larger perturbation-related changes in *P*-wave axis, duration, amplitude, or vectorcardiography-derived *P*-wave loop configuration than baseline subjects; and (4) computational or experimental SAN models incorporating heterogeneous autonomic gain, thermal gain, recovery rate, and local coupling should show reduced *R* and increased pacemaker-domain competition when dispersion exceeds coupling reserve (4, 5, 16, 17, 23–27).

Candidate readouts include PP-interval instability, short-window HRV, phase-rectified signal averaging (PRSA)-derived acceleration and deceleration capacity, transition-locked ectopic burden, and beat-to-beat *P*-wave morphology variability (28, 29).

PP-interval variability itself is not a uniquely human or necessarily pathological phenomenon, because physiological sinus arrhythmia and autonomically mediated cycle-length variability occur across mammals, including nonhuman primates (30). The present model therefore focuses on transition-locked, short-window PP-interval instability rather than baseline PP variability. Such instability should be interpreted as an indirect surface ECG surrogate of reduced pacemaker-network coherence, particularly when accompanied by *P*-wave morphology variability, pacemaker shift, atrial escape, or supraventricular ectopy.

These markers are indirect and should be interpreted as surface ECG surrogates rather than direct measurements of SAN coherence. Validation should combine high-resolution ECG or vectorcardiography with polysomnography, respiratory-event scoring, continuous temperature monitoring, autonomic phenotyping, and, when feasible, invasive or *ex vivo* high-density atrial/SAN mapping.

Recurrent orthostatic autonomic loading is treated here as a substrate-conditioning factor rather than as a discrete perturbation window. As introduced above, the relevant claim is not that human SAN heterogeneity is unique, but that a conserved heterogeneous SAN substrate may be repeatedly exposed to upright-posture-related autonomic loading (1–5, 9). In clinical studies, this substrate context should be paired with transition-linked ECG markers, including short-window PP-interval instability, *P*-wave morphology variability, pacemaker shift, atrial escape, or supraventricular ectopy, as well as phenotyping of chronic or recurrent orthostatic autonomic loading. This formulation should not be interpreted as a claim that upright posture itself is the proximate cause of SAN desynchronization.

## 8 Discussion

The present hypothesis reframes stress-related arrhythmogenesis at the level of the primary pacemaker network. Instead of treating the SAN as a fixed rhythm source whose output is merely accelerated or slowed by autonomic input, the model treats the SAN as a heterogeneous, coupled oscillator population whose stability depends on internal coherence (1–5). The proposed pathological event is a transient loss of pacemaker-network coherence superimposed on increased intrinsic-frequency dispersion and reduced coupling reserve.

This framework complements conventional electrophysiological models rather than competing with them. Membrane currents, calcium cycling, conduction, triggered activity, autonomic modulation, and source-sink balance remain central to rhythm generation and arrhythmia (1–3, 21). The present model operates at a different explanatory scale: population-level coordination within a heterogeneous sinoatrial pacemaker network (1–5, 24). Stress-related arrhythmogenesis may therefore begin before overt conduction failure or triggered activity, at the level of declining coherence among pacemaker domains.

Four observations support this synthesis. First, the SAN shows regional heterogeneity in intrinsic automaticity, ion-channel expression, calcium handling, gap-junction distribution, and pacemaker hierarchy (1–5). Second, autonomic input can modulate SAN subdomains differentially rather than uniformly (2, 3, 5). Third, autonomic control is not limited to reciprocal sympathetic-parasympathetic opposition; autonomic-space models include reciprocal sympathetic activation, reciprocal parasympathetic activation, coactivation, and coinhibition (10, 11). Fourth, abrupt physiological transitions, including stress recovery, sleep-related autonomic or respiratory events, fever, and defervescence, impose rapid changes on cardiac rhythm regulation (10–17, 21–23). Together, these observations suggest that arrhythmic vulnerability may appear when a heterogeneous pacemaker network is pushed beyond its synchronization reserve.

The upright-posture component is discussed here only as a human-relevant susceptibility context for recurrent autonomic loading, not as an independent orthostatic trigger. Mechanistically, recurrent sympathetic compensation, vagal withdrawal, later vagal recovery, or partial sympathovagal coactivation may condition adrenergically and vagally sensitive SAN domains unevenly, thereby increasing pacemaker-dispersion vulnerability in susceptible individuals. This interpretation follows the substrate-perturbation distinction introduced above and does not imply a uniquely human SAN structure.

The revised model explicitly separates slow substrate formation from fast perturbation. Sustained autonomic stress, recurrent orthostatic autonomic loading associated with habitual upright posture, chronic inflammation, fibrosis, aging, or structural SAN disease may enlarge $\sigma_{\omega }^{2}$ or reduce $K_{\mathrm{eff}}$ over days to years. Acute transitions, including stress-recovery coactivation, apnea-related arousal, hypoxemia, fever-associated thermal acceleration, or defervescence-associated thermal resetting, may then transiently increase dispersion or alter coupling on a much shorter timescale. This two-timescale structure resolves the apparent mismatch between chronic substrate-forming processes and acute physiological triggers.

In the thermal domain, the model no longer treats defervescence as a uniform cooling event. It proposes a domain-specific kinetic recovery mechanism: SAN regions with different thermal gains or recovery rates may not return to baseline in parallel during rapid defervescence. This non-parallel recovery could transiently widen intrinsic-frequency dispersion even as mean heart rate declines. The mechanism remains hypothetical, but it makes a specific experimental prediction: thermal downshifts should produce larger coherence loss when recovery rates differ across coupled pacemaker domains.

Inflammatory signaling strengthens the substrate-perturbation framework. Experimental studies indicate that local inflammatory activation can alter SAN electrical behavior and activation pattern, and sinus node dysfunction literature increasingly links inflammatory remodeling to ion-channel and structural changes (25–27). In this framework, inflammation may increase intrinsic-frequency dispersion and lower effective coupling before an acute perturbation occurs. The acute trigger then need not be extreme; a modest autonomic, respiratory, or thermal transition may be sufficient to cross the desynchronization threshold in a remodeled SAN.

The model also clarifies why SAN desynchronization may be difficult to document clinically. The proposed failure mode is transient, state-dependent, and likely to occur during short-lived transitions rather than during stable baseline recording. Surface ECG may detect only downstream manifestations such as sinus cycle-length instability, pacemaker shift, atrial escape, premature atrial complexes, or subtle *P*-wave variability. High-density atrial or SAN mapping would be required to demonstrate intranodal fragmentation directly.

Several limitations define the scope of the model. The Kuramoto formalism reduces pacemaker cells to phase oscillators and does not reproduce coupled membrane-clock and calcium-clock dynamics, nonlinear ion-channel kinetics, intracellular calcium cycling, or full action-potential morphology. Equation 1 uses a mean-field coupling term rather than a 2D or 3D anatomical lattice. Native SAN coupling is local, dynamic, heterogeneous, anisotropic, and influenced by autonomic signaling, phosphorylation, fibrosis, inflammation, and gap-junction remodeling. A future spatially explicit implementation could replace the mean-field coupling term in Eqution 1 with local heterogeneous coupling among neighboring cells or pacemaker domains. Such a lattice or network model is not implemented here. The model also omits the surrounding atrial load, even though source-sink balance and SAN-atrium exit pathways strongly influence pacemaking and conduction (31–34).

A further consequence of the static mean-field coupling assumption is that the model cannot capture state-dependent reductions or increases in local gap-junction conductance. Autonomic signaling, phosphorylation, inflammatory signaling, fibrosis, and connexin remodeling may change effective coupling over seconds to minutes or longer. If acute sympathetic activation or inflammatory signaling suppresses local coupling, the effective synchronization reserve during peak stress could be lower than Equation 1 predicts, so the model may overestimate the desynchronization threshold. Conversely, if coupling recovers or increases during post-perturbation recovery, the mean-field model may underestimate resynchronization. The single effective-coupling term also cannot represent local conduction delays, source-sink asymmetry, or unidirectional block along preferential sinoatrial conduction pathways; such spatial effects could permit pacemaker-domain competition at lower levels of frequency dispersion than a mean-field approximation would suggest.

Human SAN anatomy is particularly relevant to this limitation. Unlike a simple two-dimensional oscillator sheet, the human SAN is a three-dimensional intramural pacemaker complex embedded in the right atrial wall, with connective-tissue insulation, discrete sinoatrial conduction pathways, and redundant intranodal pacemaker regions (32, 33). Recent three-dimensional human SAN modeling further emphasizes that sinoatrial exit pathways and the SAN-atrium source-sink relationship determine whether pacemaker activity can successfully drive the atrium (34). These features may protect sinus rhythm under stable conditions, but they also imply that stress-induced coherence loss would interact with atrial load and exit-pathway selection before becoming visible on surface ECG. Human validation should therefore prioritize high-resolution mapping of pacemaker-domain competition and SAN-atrium exit behavior rather than relying on resting rate alone.

Additional limitations involve noise, parameter identifiability, and clinical validation. The present framework does not specify the statistical structure of intrinsic pacemaker noise. Biological noise in SAN cells is not adequately represented by simple additive white noise: stochastic firing variance related to calcium cycling may increase with sympathetic drive, making noise amplitude state-dependent, and temporal autocorrelation in calcium handling may introduce colored-noise structure over beat-to-beat timescales. Near the synchronization threshold, colored noise could sustain coherence-loss episodes longer than white-noise approximations predict, causing the model to underestimate the duration of arrhythmogenic windows. State-dependent noise amplification during adrenergic activation could also lower the effective desynchronization threshold relative to the deterministic formulation. These noise properties are relevant to interpretation of PP-interval micro-variability, especially spectral, nonlinear HRV, PRSA, or detrended fluctuation analysis (DFA)-based readouts, and should be incorporated in future stochastic implementations.

The latent variables representing intrinsic-frequency dispersion, effective coupling, coherence, and noise structure cannot be measured directly in routine clinical recordings. Non-invasive markers are therefore surrogate, resolution-limited, and vulnerable to artifact, respiratory modulation, autonomic variability, and atrial conduction changes. These limitations support interpreting the framework as a reduced phenomenological model rather than as a quantitatively calibrated clinical tool.

The proposed concept also differs from, but may overlap with, classical wandering atrial pacemaker. Wandering pacemaker describes an ECG-level sequential shift in the dominant atrial pacemaker, usually inferred from changing *P*-wave morphology. SAN desynchronization, as used here, denotes loss of phase coherence or partial fragmentation within the pacemaker population before a single dominant exit site becomes clinically obvious. This distinction remains conceptual until validated by high-density mapping capable of resolving simultaneous or near-simultaneous pacemaker-domain competition.

Despite these limitations, the model integrates established SAN heterogeneity, differential autonomic responsiveness, stress-recovery sympathovagal coactivation, sleep-related autonomic and respiratory perturbations, recurrent orthostatic autonomic loading as a substrate-conditioning context, thermal sensitivity of pacemaker mechanisms, and inflammatory remodeling into one testable framework (1–17, 21–34). Its central claim is not that stress-induced SAN desynchronization has already been proven in humans. The claim is that pacemaker coherence provides a plausible and measurable systems-level dimension of rhythm stability.

Future validation should proceed across computational, translational, and clinical levels. Spatially explicit human SAN models should incorporate local coupling, dynamic coupling modulation, anisotropy, atrial load, regional pacemaker domains, heterogeneous autonomic and thermal gains, sinoatrial exit pathways, local conduction delays, and state-dependent colored noise. Experimental preparations should test whether autonomic, inflammatory, or thermal perturbations increase functional separation among superior, central, and inferior pacemaker regions, alter SAN-atrium exit behavior, or produce transient local conduction delay or block. Clinical studies should pair transition-locked electrocardiographic markers with sleep staging, respiratory events, temperature, autonomic measures, and phenotyping of chronic or recurrent orthostatic autonomic loading.

This framework should therefore be interpreted as a mechanistic hypothesis suggesting that attention should also be directed from rate control alone toward coherence control within the primary pacemaker network. If validated, this hypothesis may help explain why arrhythmias often cluster during abrupt physiological transitions despite unremarkable resting evaluation, and may motivate future risk assessment based on dynamic synchronization reserve rather than baseline sinus rate alone.

## 9 Conclusion

This article proposes a systems-level hypothesis in which stress-related arrhythmogenic vulnerability arises from reduced synchronization reserve within a heterogeneous SAN pacemaker network. Sustained autonomic stress, recurrent orthostatic autonomic loading associated with habitual upright posture, inflammatory remodeling, fibrosis, or structural SAN disease may increase intrinsic-frequency dispersion or reduce effective coupling, whereas acute autonomic, respiratory, or thermal transitions may expose this vulnerable substrate. Thus, upright posture is framed as a chronic human-relevant substrate-conditioning context, not as a discrete acute trigger. The model does not replace ionic, conduction-based, reentry, or triggered-activity mechanisms; it adds pacemaker coherence as a complementary dimension of rhythm stability. Clinically, the hypothesis predicts transition-linked sinus cycle-length instability, supraventricular ectopy, pacemaker shift, and *P*-wave morphology variability. Validation will require spatially explicit SAN models and transition-locked ECG, autonomic, sleep, temperature, and mapping studies.

## Data Availability

The original contributions presented in the study are included in the article/Supplementary Material, further inquiries can be directed to the corresponding author.
