# Supplementary figures and images for: Stress-induced pacemaker desynchronization in the sinoatrial node
Source: Front Cardiovasc Med. 2026 Jun 23;13:1852965. doi: 10.3389/fcvm.2026.1852965 (PMC13337363; doi:10.3389/fcvm.2026.1852965)

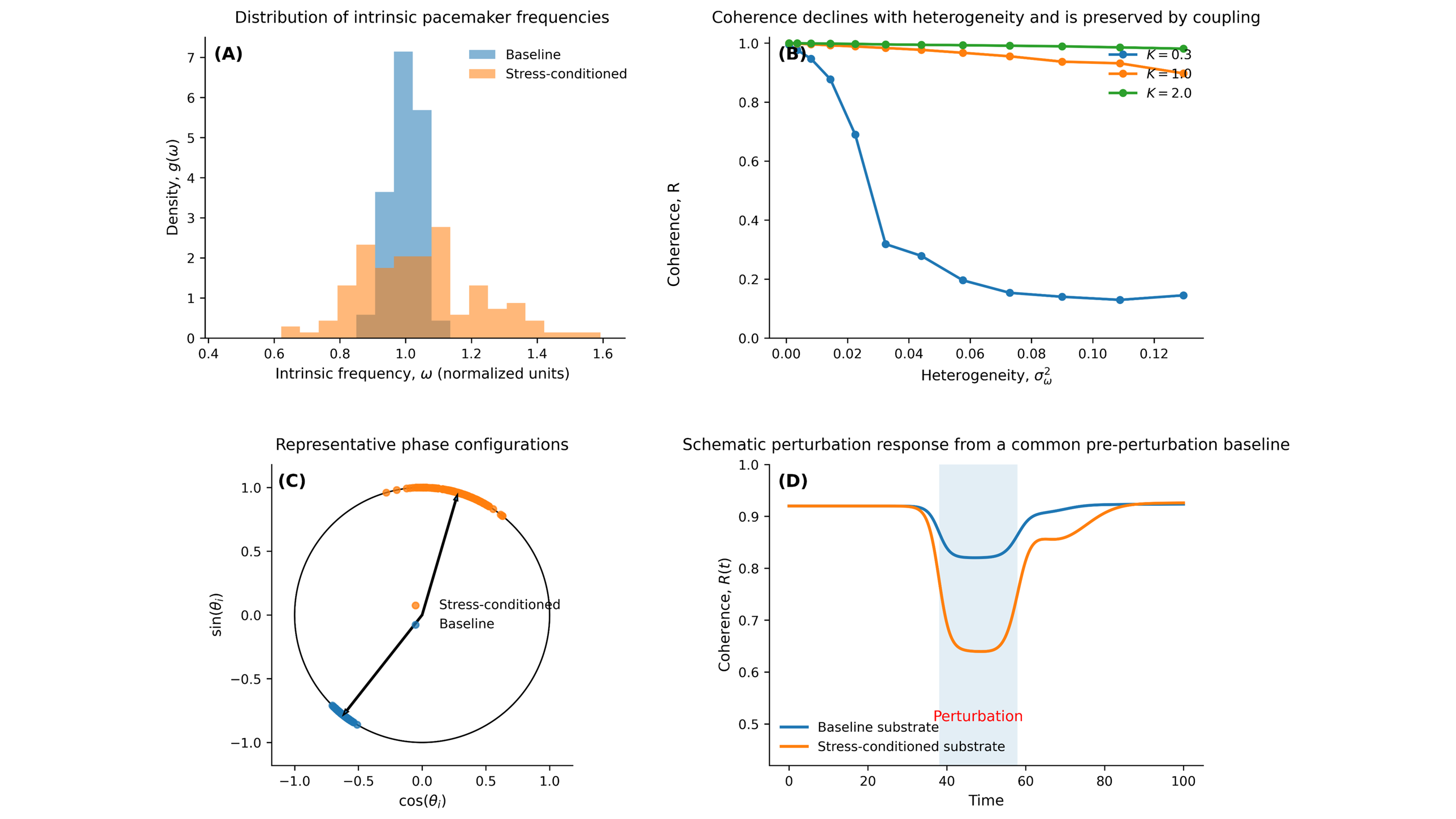

Supplement: Supplementary file 1 [file Datasheet1.zip › Figure1.tif]
